# Articulations of ‘Funeral’ in Swedish Newspapers During the Covid-19 Pandemic

**DOI:** 10.1177/00302228231174601

**Published:** 2023-05-10

**Authors:** Karin Jarnkvist

**Affiliations:** 1 Department of Humanities and Social Sciences, 6311 Mid Sweden University, Sundsvall, Sweden

**Keywords:** Covid-19, funerals, newspapers, CDA, intersectionality, Sweden

## Abstract

This article explores how ‘funeral’ was articulated in Swedish newspapers during the Covid-19 pandemic and how such articulations relate to power and ideology. Articles from the six most prominent Swedish newspapers, published over 2 years, have been analyzed using critical discourse analysis and intersectionality. The study reveals three funeral discourses dominating during different periods of the pandemic: ‘Funeral as a risk,’ ‘Funeral as an essential ritual,’ and ‘Funeral as a profession.’ Altogether, the three discourses expose an ideal of ‘the responsible mourner.’ This rational woman follows the funeral restrictions and arranges a church funeral shortly after the death of a relative. The ‘good funeral’ is portrayed as a church funeral with physically present mourners, performed according to the deceased’s will and in honor of the dead. The ‘bad funeral,’ described as the opposite of the ‘good funeral,’ dominates the understanding of the pandemic funeral situation.

## Introduction

Funeral is seldom a subject of interest in Swedish news media, except in crises (Reimers, 2003), such as the coronavirus pandemic in 2020–2022. The fast development of the disease Covid -19, with the deaths of thousands of swedes, made funerals and grief central subjects in media reports during these years. There were also many regulations regarding funerals which affected funeral practices in several ways (i.e., Simpson et al., 2021). This article explores how ‘funeral’ was articulated in Swedish newspapers during the coronavirus pandemic and how such articulations relate to power and ideology. Articles from the six most prominent Swedish newspapers, published over 2 years, have been analyzed using Fairclough’s critical discourse analysis (CDA) (Fairclough, 1992; 2010). Intersectionality has been used as an analytical tool in the analysis.

Although funeral research is extensive, studies on representations of ‘funeral’ in news media are rare. However, media dramatically impacts people’s way of thinking nowadays. Research on how newspapers portray funerals is needed to understand norms regarding death practices (Selman et al., 2021). So is the use of intersectionality, which is seldom considered in previous funeral studies. The intersectional perspective may uncover hidden power structures and bring unequal relations between groups of people to light. That is important for making society’s practices regarding death and bereavement more equal.

While previous media studies during the Covid-19 pandemic focus on short periods of the pandemic (i.e., Selman et al., 2021; Sowden et al., 2021), this paper examines articles from March 2020 to May 2022, which makes it possible to grasp changes in how ‘funeral’ was articulated in news media during the whole period of the pandemic.

This study reveals three main discourses on ‘funeral’ in the analyzed newspaper articles: ‘Funeral as a risk,’ ‘Funeral as an essential ritual,’ and ‘Funeral as a profession.’ Moreover, constructions of the ‘responsible mourner,’ the ‘good funeral,’ and the ‘bad funeral’ are done in the newspaper articles and revealed in the study.

The article is structured as follows: First, I overview previous research and the study’s theoretical point of departure. After a description of the material and method used in the study, the result and analysis are presented. Finally, I discuss the findings.

## Background

When Covid -19 spread worldwide in 2020, it rapidly changed practices concerning death, including funerals. Studies have investigated ‘new’ death ritual practices (i.e., MacNeil et al., 2021), changes in the funeral industry (i.e., Turner & Caswell, 2022), and different aspects of effects on bereavement caused by funeral restrictions (i.e., Simpson et al., 2021). Media studies have focused on the use and importance of digital media for bereaved people (Asgari et al., 2022). A few studies have examined grief and bereavement in news media (Sowden et al., 2021; Selman et al., 2021). These studies often touch on funerals but are seldom the main subject.

However, a few previous studies focus on funeral articles in the UK newspapers. Funerals were most often treated in features, within a fear narrative relating to funeral restrictions, or in a framework of tragical stories with interviews of bereaved people who had lost their loved ones (Sowden et al., 2021). In the articles, funerals were often portrayed as absurd, as there were strict regulations around them. The newspapers focused on what was forbidden rather than permitted and offered hardly any practical help to mourning people (Selman et al., 2021). Selman et al. (2021) found a shift in focus in articles on funerals during different periods of the Covid-19 pandemic. At the pandemic’s beginning, the newspapers wrote about dramatic examples from Italy and America with suggestions for making funerals in the UK. Some weeks later, the articles focused on personal stories of death and funeral planning. Funerals were described as an essential help in the grief process.

### The ‘Good Death’ and Neoliberal Ideology

Even though studies on the construction of ‘funeral’ in media are rare, research on death and bereavement in media is extensive. Research has shown that media has a central role in the understanding of death and bereavement by representing ideas of ‘good deaths’ (Van Brussel & Carpentier, 2012), ‘bad deaths’ (Nelson-Becker & Victor, 2020), grief and bereavement (Selman et al., 2021). Media creates sense-making narratives and discusses and imposes cultural ideas about death and bereavement (Hanusch, 2010). However, what then is considered a ‘good death’? According to the ideology of ‘the good death,’ death should be met in the presence of loved ones, and dying should follow the individual’s will (Simpson et al., 2021). During the pandemic, the idea of the ‘good death’ was contested, and the media presented images of a ‘bad death.’ Dying alone, without control and family support, was portrayed as a nonnormative event; the lonely death was seen as a death that matters, and where people die alone, societies should respect it and learn from it (Nelson-Becker & Victor, 2020). The ideal of the ‘good death’ is in line with the neoliberal ideology of individual responsibility (Foucault, 2008). According to Foucault (2008), individual freedom, which is the landmark of neoliberalism, is used as a disciplinarian force. Foucault uses the term ‘governmentality’ to describe how discipline is achieved today, not by traditional hierarchical power but by creating through discourse. These boundaries limit and constrict choice while instantaneously disseminating individual agency and free will.

### Critical Discourse Studies and Intersectionality

This study is informed by Critical Discourse Studies (CDS), which investigate social inequality as it is articulated, represented, legitimized, and so on by language use (Wodak & Meyer, 2015, 12). Drawing on CDS, and especially the perspective of Fairclough (1992, 2010), I understand discourse as a mode of action, a way for people to act upon each other and upon the world, and a representation (c.p. Wodak & Meyer, 2015). Discourse contributes to the construction of ‘social identities’ and ‘subject positions’ for all individuals; it is involved in constructing social relationships between people and constructing knowledge and belief systems (Fairclough, 1992, 64), such as norms and values about funerals. In other words, a particular discursive event, such as articulations of ‘funeral’ in news media, is shaped by but also shaping, the situation(s), institution(s), and social structure(s) that frame it. In line with Fairclough et al. (1997), I understand discursive practices to have an essential ideological effect by producing and reproducing unequal power relations between (for example) gender or ethnic/cultural majorities and minorities through how they describe things and position people (c.p. Fairclough et al., 1997, 258).

I have used intersectionality as an analytical tool in processing the material. According to intersectional perspectives, social stratifications affect people’s living conditions and are also remade by human actions. One stratification cannot be separated from other stratifications but relates to them and is affected by them (Brah & Phoenix, 2004). Intersectional perspectives can uncover positions of privilege that are often ignored or taken for granted and reveal how power structures and positions are challenged and renegotiated. Therefore, intersectional analyses are appropriate when studying the complexity of a social phenomenon, such as the articulation of funerals in Swedish newspapers. As the analysis will show, one of the leading discourses on funerals in Swedish newspapers during the pandemic was the discourse of funerals as a risk. In my interpretation of the articles, I use intersectional risk theory (Giritli Nygren et al., 2019), which departs from intersectional theory and provides tools for understanding responsibility, vulnerability, and the decision-making power of individuals and groups can be attributed to social structures. According to intersectional risk theory, ‘risks are performative and are always co-articulated with norms and discourses, which, in turn, impact how (in)equalities are and can be performed or “done”’ (Giritli Nygren et al., 2019, 25). Risk is a way of, i.e., ‘doing’ class, gender, ethnicity, and vice versa. According to intersectional risk theory, risk can be distinguished from what poses a risk, such as Covid-19. Risk is the social construction of the probability of being sick in, for example, Covid-19.

## Material and Method

I conducted a document analysis of online newspaper articles exploring how the Swedish news media portrayed funerals during the coronavirus pandemic. Six newspapers in Sweden were selected, representing two of the most extensive morning papers (Dagens Nyheter (DN), which is independent/liberal, and the liberal/conservative Svenska Dagbladet (SvD)), the two most extensive tabloid papers (the social democratic Aftonbladet, and Expressen, which is liberal), and the two most extensive regional papers (the liberal Göteborgs-Posten (GP), and Sydsvenskan, independent/liberal). These publications cross-readership demographics and the political spectrum and represent various newspaper types. The sampling included different genres of newspaper material: opinion texts (editorials, op-eds), news articles, features, and cultural reviews. The analyzed media texts were published during the coronavirus pandemic, which lasted for 2 years, from March 2020 to May 2022.

I used the news archive program Mediearkivet/Retriever when searching for relevant newspaper articles, using the following keywords as searches: Covid/corona and funeral. Searches were completed for a timespan covering the period when Covid-19 was regarded as a pandemic (2020-03-12 – 2022-05-12). I included articles from specified news sources mentioning funerals in relation to the COVID-19 pandemic. The criteria for including an article in the analysis were that the word “funeral” should be mentioned in the article and that “funeral” as an occasion was given a function in the article. In some articles, “funeral” was mentioned once in a sentence, while others focused on the rite. I excluded articles discussing funerals unrelated to COVID-19 or just mentioning the word funeral but not giving it a function in the text. The search yielded 65 relevant articles. For a full view of the material, see Appendix 1.

### Analysis

I analyzed articles during the whole pandemic period to encompass possible changes in reporting of funerals during the COVID-19 pandemic. My analysis envisioned ‘funeral’ as a social construct and was inductive, aiming to identify and understand the discourses on funerals within the newspaper material. My starting point was that newspaper discourses are not neutral. On the contrary, they impact how bereavement is experienced and what is understood as ‘normal’ about funerals. In the analysis, I employed a model informed by Fairclough’s three dimensions of analyzing discourses in texts: a descriptive phase, a processing phase, and an explaining phase (Fairclough, 2010, 132 pp.). In the first (descriptive) phase, I identified the main 'themes' in the funeral framing. Coding focused on manifest and latent themes and trends/differences across time and publications (see further in Fairclough, 1992, 118p.). Manifest themes are the types/aims of articles and how funerals are presented and portrayed, for example, funerals being framed as risks for spreading Covid-19 disease. Latent themes are about the implicit content, the use of metaphors/symbols, and contradictions in the text, such as the use of Christian symbols and the taken-for-granted Lutheran funeral. I also analysed trends/differences across time and publications. For example, the framing of funerals changed during the pandemic relating to the spread of the disease, and some newspapers framed funerals as a risk more than others.

In analyzing texts, one is always addressing questions of form as well as questions of meaning. I have analyzed wording from the perspective of alternative wordings and their political and ideological significance, as well as word meaning and metaphors (see further in Fairclough, 1992, 76, 185). In the analysis, I also paid attention to what was not expressed in the texts and what was taken for granted.

In the second phase (processing), I considered intertextuality in interpreting how context and interests influenced the articulations of funerals. Using intersectionality as an analytical tool, I analyzed the funeral articulations identified in phase one in terms of how they related to the most prominent power structures in the material: gender, religion, and social class. Finally, in the third (explaining) phase, I analyzed the funeral articulations from the perspective of how they linked to specific ideologies and discourses. Through this three-step analysis, I identified recurring narrative patterns and themes within and across the articles and considered these relating to social theories. I have ensured the trustworthiness of my analysis by including all relevant articles during the pandemic period in the analysis, making the included articles readable by listing them (see Appendix 1) and being transparent with how I have conducted the analysis of the material. The articles were all written in Swedish, and the quotes presented in the article have been translated.

## Findings

Three funeral discourses are revealed in the material: ‘Funeral as a risk,’ ‘Funeral as an essential ritual, and ‘Funeral as a profession.’ There are also themes within each discourse. The discourses intersect with different discursive power categories and are influenced by neoliberal ideologies. Below, I present my analysis of each discourse.

## Funeral as a Risk

The discourse of ‘funeral as a risk’ dominated the rhetoric in media during the first months of the Covid -19 pandemic, in March and April 2020, and the late autumn of 2020 when the government decided to have new limits on how many people were allowed to attend funerals. The discourse is constructed in three ways: by describing funerals as events where the virus is spreading, by informing about and discussing funeral regulations, and finally, by relating to a narrative about the difficult situation of the pandemic. ‘Funeral’ intersects with gender, religion, and social class and is closely related to the neoliberal ideology of individual responsibility. In this case, individuals are seen as responsible for preventing risks related to funerals (c.p. Foucault, 2008). The narrative of the funeral as risk is primarily present in the tabloids Expressen and Aftonbladet (15 articles), followed by the regional newspapers Sydsvenskan and GP (seven articles) and the national newspapers SvD and DN (seven articles).

### Risk of Infection

At the begging of the pandemic, attending a funeral was primarily seen as a risk of infection by Covid-19. This way of interpreting ‘funeral’ is uncommon in times of no pandemics. The situation makes clear that an event, such as a funeral, does not become a risk until it is interpreted as such. The socio-spatial-temporal context determines what is considered a risk (Giritli Nygren et al., 2019). At the beginning of the pandemic, there was little knowledge of whether the virus was contagious even after a person had died of the disease. The dead bodies were covered uniquely, and the coffins were not opened. The newspapers wrote about the risk of becoming infected at funerals, famous swedes being ill or dying of the disease, and people being afraid of attending funerals because they could get infected by other participants. Under headings like ‘Cancel of fear’ (Aftonbladet 2020-03-29) and ‘The virus forces new routines for funerals’ (DN 2020-03-25), representatives of the Church of Sweden and funeral homes are interviewed about the fear that they experience that many relatives feel, and how they treat the funeral restrictions in their organizations. The word ‘fear’ is frequently used in the articles.

Individual funeral responsibility is emphasized, and the interviewed representatives are portrayed as responsible citizens, making the funeral as secure as possible. One of the most robust articulations of individual responsibility is an article about a family that had planned to have a big funeral for the mother of the family, who died of cancer at the beginning of March 2020. The ingress says:

The family had planned for [name] funeral in detail and expected hundreds of guests. The corona infection means many mourners must now follow the funeral online instead. - We cannot have it on our conscience to gather so many people in the church, the husband [name], senior physician in bacteriology and virology, says. (Sydsvenskan 2020-04-02)

In the ingress, the husband talks about it being unresponsible to gather many people in church during a pandemic. By doing so, the newspaper positions the husband as a responsible person. Following Foucault (2008), I understand the concept of ‘risk’ as primarily used to make people follow specific regulations. By mentioning the husband’s medical doctor profession, the newspaper signalizes that this man knows what he is talking about. Individual responsibility intersects with social class. The same is done in several articles (Aftonbladet 2020-11-27 a and b, Expressen 2020-11-27, GP 2020-11-27) in late November 2020 reporting about the Swedish prince and princess being infected by Covid-19 after attending a family funeral. All articles quote the information manager of the royal house, emphasizing that all those who attended the funeral had been overly cautious, kept a social distance in church, and were tested negatively before the funeral. Despise all that; it seems like the prince and princess had become infected by Covid-19 at the funeral. The message is that the disease spreads exceptionally quickly and that the couple acted responsibly but were infected anyway.

Individual responsibility is also constructed in intersection with religion/non-religion. In an article in Aftonbladet (2020-03-29), an interviewed Muslim woman, who goes against Muslim traditions and cremates the body of her dead father, is positioned as a responsible citizen as she ‘prioritizes the living before traditions,’ as she says in the article. ‘Secularity’ is the norm, while Islam is portrayed as a recalcitrant tradition. My interpretation is that medical knowledge, respect for restrictions, and secularity, are expressions of what is considered rational in the current situation. With this understanding, the ‘responsible citizen’ acts ‘rationally’ and prioritizes others’ security before their own emotions or religious traditions (c.p. Author, coming).

### Following Regulations

During the pandemic, the primary responsibility is directed toward individuals in terms of following the social regulations of the government concerning funerals. The analyzed articles report the rules that shifted during the pandemic but also make space for criticism of the restrictions. For example, in Aftonbladet (2020-12-13), the news columnist writes about the absurdity (as he sees it) of letting thousands of people shop at the malls. In contrast, only a few people are allowed to attend funerals.

In November 2020, the government decided that only eight persons were allowed to come together at funerals and on other occasions. Representatives from different religious congregations criticized the decision, with the Church of Sweden in the front (Aftonbladet 2020-11-20). After that, the government changed the restrictions for funerals and allowed a maximum of 20 persons on funerals, which several newspapers wrote about (Aftonbladet 2020-11-20, SvD 2020-11-21, Sydsvenskan 2020-11-21). In these articles, the Church of Sweden is portrayed as an actionable church, standing on the side of the people. However, the situation is another for Muslims in Malmö, who are portrayed as irresponsible, as there had been too many people attending funerals on several occasions, and police had been called to the cemetery to bring order (Expressen 2020-12-30). An alarming and conflicting language is used in the article, with words like ‘forced’ and ‘threatened,’ and Muslims are positioned as ‘problematic’ (see further in Author, forthcoming).

### Funerals as a Worst-Case Scenario

In some articles, funerals are discussed, sometimes only mentioned, as an example of a worst-case scenario. The word ‘funeral’ signalizes the seriousness of the situation and actualizes the risk of dying in Covid-19. The death rates were higher in some residential areas than others, and people were interviewed about how many funerals they had attended, sending a message that the disease can cause the death of many people.

Older adults living in elderly homes are positioned as especially vulnerable. In interviews, relatives of people who have died of the disease criticize the elderly care for not acting responsibly. At the end of one of the articles in GP, one can read: ‘The daughters are now busy planning the funeral and mourning their deceased mother. [Name] finds it important to tell so that others will know how bad it can get’ (GP 2020-04-17). Mentioning the funeral indicates that ‘the worst thing’, death, happened.

## Funeral as an Essential Ritual

However, funerals are not only regarded as a risk. During the pandemic, the funeral is also portrayed as an essential but restricted ritual for bereaved people. The discourse is constructed in three ways: ‘the restricted ritualization of death,’ ‘the funeral as a part of the mourning process,’ and ‘the funeral as a relation.’ ‘Funeral’ is constructed in intersection with religion and gender and is closely related to the ideology of the ‘good death,’ according to which death should be met in the presence of loved ones and the process of dying should follow the will of the individual (i.e., Cottrell & Duggleby, 2016; Nelson-Becker & Victor, 2020; Simpson et al., 2021).

The articles are written in informal language. Quotations are often used as headings, and the interviews are written in a way close to how people speak in ordinary conversations. Conversational discourse is, according to Fairclough, linked to the use of expert systems in the public domain, such as media (Fairclough, 2010, 99), something that is prominent in the analyzed material, where ‘experts’ on grief and funerals often are interviewed. Funeral as an essential ritual is primarily present in the tabloids Expressen and Aftonbladet (13 articles), followed by the national newspapers SvD and DN (10 articles), and finally, the regional newspapers Sydsvenskan and GP (five articles).

### The Restricted Ritualization of Death

At the beginning of the pandemic, the problem of not being able to have a funeral is dealt with in the newspapers, often from a therapeutic perspective and related to the discourse of funeral as a risk. In all articles on this theme, the funeral is described as a necessary ritual for being able to ‘say goodbye’ to the dead person. Primarily female relatives of deceased men are interviewed about the difficulty of dealing with the death when they have not been able to make the funeral the way they wished or maybe not been able to attend the funeral at all because of the restrictions. The daughter of a man who died in Covid-19 says:

It is strange, somehow, dealing with the demise. I have not even been able to attend the funeral, so I have never said goodbye for real, in place. (Expressen 2021-01-03)

The norm of a funeral with physically present mourners is revealed in the quotation. The interviewed woman, who is a nurse, meets patients infected by Covid -19 in her daily work. She expresses anger when talking about those who do not seem to care about the restrictions, as she says this will make the infection spread even more. The mix of anger and grief makes the article very emotional. In another article, a woman talks about the loss of her husband and her experiences at the live-streamed funeral (SvD 2021-08-23). One of the friends of the deceased describes how he attended the funeral at a distance, and a researcher and a psychologist are interviewed as 'experts' on grief and social media. A therapeutic discourse frames the articles. ‘Counselling,’ the ‘apparently non-directive, non-judgmental, empathizing way of talking to people one-to-one about themselves and their problems' (Fairclough, 2010, 66), is a frequently used technique in many articles. The article’s overall message is that the deceased died a ‘bad death’ because of the pandemic, alone or at a hospital, without control, isolated from their relatives (c.p. Cottrell & Duggleby, 2016). In line with the ideology of the ‘good death,’ funerals during the pandemic are primarily constructed as ‘bad funerals,’ as they are without mourners present, or at least with only a few mourners present.

‘Funeral’ is, in some cases, also used as a signal word. In interviews, people whom the disease had infected might say, ‘I started to plan for my funeral,’ which signalizes that the person believed that his/her end of life was near (i.e., Aftonbladet 2020-12-03). The expression of planning one’s funeral goes hand in hand with the neoliberal ideology of individual responsibility and the idea of the ‘good death’ as control (c.p. Cottrell & Duggleby, 2016).

### Funeral as a Part of the Mourning Process

The funeral is also presented as an essential part of mourning, as it is a moment of ‘saying goodbye’ and honoring the dead person. Priests, therapists, funeral directors, and other professionals talk about the importance of ritualization for people with grief. These articles are an outcome of today’s reflexive society (c.p. Giddens, 1991). The individual is supposed to need an ‘expert’ to understand him/herself, in this case, the troubles one might have with grief and mourning during the isolating pandemic. The ‘experts’ talk about the importance of being physically present in the same room as the dead person during the funeral, as it is said to make the bereaved understand what has happened. In one such article, the interviewed psychologist says:

Even if we are prepared, it sometimes takes a while to fully understand that the person is dead. We need proof and see that the person has left the body. (DN 2020-05-05)

The new situation, with funeral restrictions, is discussed as a problem in the articles. Digital funerals are often mentioned as a possibility to deal with the new situation. However, sometimes they are also critically discussed. The Luther Christian framework dominates, as several priests are interviewed as 'experts' in the articles. Like other ‘experts,’ the interviewee also talks about the importance of sharing the moment with others, as it is told to help people not feel lonely. One of the articles (DN 2020-05-05) includes phone numbers and web addresses for non-confessional help organizations and the Church of Sweden’s helpline. However, there is no contact information for other religious organizations. Again, Lutheran Christianity and secularity are made the norm.

### The Relational Perspective of Funerals

In some articles, funerals are framed by a narrative about close relationships among family members and friends. The articles deal with difficulties with saying no to people who wish to attend the funeral or cannot hug each other during funerals. Like in the UK newspapers (Selman et al., 2021), the impact of funeral restrictions is described as unjust. The message is that the deceased should have the right to have a ‘good funeral,’ that is, a funeral in church with many mourners physically present and where the deceased is honored. Also, here, the therapeutic discourse is emphasized in the articles. Some articles discuss the increase of new registrations in archives where one can register information that one’s relatives would need to have after one’s death, for example, wishes about the funeral (DN 2020-04-19, Sydsvenskan 2020-12-19). The registration is described as something positive and interpreted as a sign of caring for one’s relatives. It can also be seen as an expression of the ‘good death’ as control (c.p. Cottrell & Duggleby, 2016).

Cremation and burial without a registered funeral, so-called ‘direct cremation,’ have increased in Sweden since 2014 and even more during the pandemic. The situation is discussed in an article in Aftonbladet under the heading ‘A final farewell - without a funeral’ (2020-12-14). The head of Sweden’s funeral directors' association is interviewed about the shift of burial culture in Sweden. The change is described as a generational issue, with people born in the 60s and 70s not having time to arrange and attend funerals. However, the interviewee no doubt wishes that more people should choose to have funerals for their deceased relatives. No contradictory ideas are mentioned in the article. Consequently, funerals are portrayed as the norm, and people who choose not to have an ordinary funeral are positioned as selfish, not prioritizing their dead relatives. This interpretation of ‘direct cremation’ is also presented in an article in DN (2020-12-26), in which the archbishop of the Church of Sweden is interviewed about the ritualization of death which she, according to the article, finds to be of particular importance, but also neglected, in a time that ‘rewards speed, mobility, and individuality,’ as she says.

## Funeral As a Profession

At the end of spring 2020, the newspapers started to report on funerals from the perspective of death- and funeral professionals. As many people at the beginning of the pandemic chose not to bury or cremate their dead relatives, there was a problem with storing dead bodies. The message in the articles was that relatives of the deceased needed to hurry up with the funerals. The articles about storing problems are followed by features about busy workdays and the heavy workload of those working with death and funerals. The article forms are mainly interviews with professionals and features from working places. ‘Funeral’ is constructed in intersection with gender and religion. The discourse is framed by a hero narrative and inspired by the neoliberal ideology of the responsible citizen and the ‘good death’ ideology.

Funeral as a profession is nearly exclusively present in the national newspapers SvD and DN (seven articles), followed by the regional newspapers Sydsvenskan and GP (one article), and finally, the tabloids Expressen and Aftonbladet (no articles).

### The Urgent Funeral

The first article about the experienced problem of storing dead bodies was published in SvD at the end of April 2020. One of the lines in the ingress says: ‘One problem is that relatives wait to hold funerals’ (SvD 2020-04-22). The article also says that one of two aggravating circumstances with the handling of the deceased in Stockholm is if relatives of the deceased do not follow the funeral law, which states that one has 30 days to bury the body or carry out the cremation. At the end of May 2020, the government appealed to mourners not to postpone funerals. DN wrote:

The Minister of Culture and Democracy, Amanda Lind, urges relatives not to postpone funerals because it puts severe pressure on the country's funeral business. (DN 2020-05-26)

Expressions like ‘urges’ and ‘severe pressure’ indicate that the situation is serious and that to be a responsible citizen, one must take care of the bereaved relatives and have a funeral. Moreover, in both articles, it is taken for granted that relatives will have a funeral for the deceased, even though the law does not say anything about having a funeral. Around 10% of those who died during the pandemic were buried without a known funeral.

### The Funeral Working Hero

The narrative of the urgent funeral connects to the narrative of the funeral working hero. Features of funeral workers' (primarily women) daily work during the pandemic describe exceptional working conditions that suddenly have become ordinary days in a funeral worker’s life. The high death rate in the population makes the workload massive. With headings like ‘Funerals increased by 261% in April’ (DN 2020-05-26) and ‘30% more deaths compared to last year’ (GP 2020-05-22), funeral directors and representatives from different religious congregations (mainly the Church of Sweden) are interviewed about their busy work. In the case that representatives of other religious communities are interviewed, they are exotified in a way that representatives of the Church of Sweden are not. In an article about a morgue in Gothenburg, the unit manager says:

It has been stressful; we have worked very hard and had to bring in temporary workers if necessary. It was challenging to get hold of protective equipment for a while, but it worked out. Moreover, there is a great sense of responsibility among those who work here. (GP 2020-05-22)

Like the nurses, people working with death are portrayed as very loyal and dedicated to their work, which is often described as a vocation (c.p. Mohammed et al., 2021).

Another perspective of the funeral working hero is the portrayal of the undertaker who has a funeral without mourners. Under the heading ‘More and more are dying and being buried alone’ (DN 2020-12-26), a funeral with no living person present except the undertaker is featured. In the article, so-called ‘direct cremation’ is discussed and, in this case, interpreted as an aspect of greater loneliness in Sweden. The undertaker says that she, by having the funeral even though no mourner is there, represents all those who knew the deceased. ‘It feels like a nice mission,’ she says. The undertaker is positioned as a responsible citizen who cares for and honors the deceased, in contrast to all those who, according to the article, do not seem to care for their relatives by arranging funerals.

## Discussion

This study examines Swedish newspapers’ articulation of ‘funeral’ during the corona pandemic 2020-2022. The critical discourse analysis reveals three funeral discourses: 1) ‘funeral as a risk,’ 2) ‘funeral as an essential ritual,’ and 3) ‘funeral as a profession.’ The discourse of ‘funerals as a risk,’ most common in media at the beginning of the pandemic, is framed by a neoliberal ideology emphasizing individual responsibility. Following Foucault’s (2008) perception of risk as a form of power-wielding, framing funerals as risk events can be seen as governing bereaved peoples' behaviors. The language used in the articles is often framed by a narrative of fear and conflict and linked to sensationalism, something also recognized in research on funerals in UK newspapers (Sowden et al., 2021). The rhetoric may have caused additional terror and fear of death among readers. I agree with Sowden et al. (2021) when they argue that the media should have been able to calm the readers instead of making them more frightened if they had used more neutral language. This should probably have been more helpful for many people.

Risk is done at the intersection of social class and religion. Well-educated and/or high-class people, representatives of the Church of Sweden, and people positioned as ‘secular’ (or ‘rational’) are, to a great extent, considered responsible individuals, while, for example, Muslims, following religious funeral traditions, are portrayed as irresponsible. A similar pattern is revealed in a study on media reports on religion in Australia (Halafoff et al., 2021), where Muslim, Jewish, and some ethnic Christian religious minorities got negative media attention and were accused of deviance in not following Covid-19 restrictions.

The discourse of ‘funeral as an essential ritual’ is linked to the discourse of ‘funeral as risk.’ At the beginning of the pandemic, the newspapers wrote about the problematic situation of not being able to have a funeral at all, or at least not the way people wanted, because of the restrictions. However, funerals are also described as rituals that need to be done to be able to deal with one’s grief, and many articles discuss funerals from a relational perspective with a focus on the funeral as an occasion to gather family and friends and mourn together, a need which was not possible to meet because of the restrictions. The idea of the funeral as a vital part of the grieving process is also recognized in UK newspapers (Selman et al., 2021).

A therapeutic discourse with informal language frames the articles. Informal language can be seen as a strategy to eliminate overt power markers by making the articles like private conversations (c.p. Fairclough, 1992, 204). By using ‘counseling’ as a technic in the articles, aspects of people’s private lives are subtly drawn into the domain of power (the media) (c.p. Fairclough, 2010, 66). This technique is in these articles related to gendered power relations. There are almost exclusively women who are interviewed as relatives of deceased men. The dead relatives are often fathers of the interviewed women but sometimes also brothers or husbands. By letting women talk about the grief and loss of their loved ones, women are consolidated as the ones who keep the family together, are reliable when it comes to family matters, and are responsible for taking care of everything after death within the family. The overrepresentation of women as interviewees also confirms the image of women being good at talking about feelings. The portrayal of the mourning woman talking about the loss of a loved man keeps the ‘heterosexual matrix’ stable (c.p. Butler, 2006).

Within the discourse of ‘Funeral as a profession,’ ‘funeral’ is not only normative but also positioned as something needed to be taken care of shortly after the death of a relative. Undertakers (mainly women) who conduct funerals without mourners are portrayed as responsible citizens who honor the deceased even though no one who knew the deceased while alive seems to care about the dead. In the article in DN (2020-12-26), the interviewed undertaker appears to understand her role as a representative for all who knew the deceased in life. This way of understanding the act is known from previous research on undertakers. However, so is also a feeling among undertakers of being obliged to present, as they see it, an inauthentic account of the deceased at these funerals (Turner & Caswell, 2022). In this way, the positioning of undertakers as heroes might be more of a burden than an incentive for funeral professionals. I agree with Mohammed et al. (2021), who, in their study on the discourse on nurses as heroes, argues that ‘the hero discourse is not a neutral expression of appreciation and sentimentality, but rather a tool employed to accomplish multiple aims.’ Regarding funeral workers, the aim might be the normalization of their performance of model citizenship and femininity, as well as supporters of the ‘good funeral,’ which is understood as a funeral with physically present mourners.

The ideology of the ‘good death’ is closely related to the ideology of neoliberalism and the ideal of individual responsibility. The embracement of the planning for one’s funeral, which is emphasized in several articles, relates to the ‘good death’ as control (c.p. Cottrell & Duggleby, 2016), as it speaks to neoliberal political and social values of autonomy, freedom, and choice, and evokes Foucault’s notion of governmentality (c.p.Foucault, 2008). The individual ‘freedom’ is accessible only to a certain extent as it does not cope with spontaneity or ‘uncontrol.’ Relatives who choose not to have a funeral are regarded as egoistic, and the deceased buried without a funeral are positioned as lonely. Those who plan for their funeral in advance, on the other hand, are regarded as responsible citizens. The planning is interpreted as an act of care and love for their relatives, who will take care of the funeral after the person’s death. Planning also makes work easier for people working with funerals and might be another reason why it is appreciated among funeral professionals. Maybe the increase of people planning for one’s funeral also can be seen as a reaction to the rise of ‘direct cremation.’ The one who plans one’s funeral can ensure that there will be a funeral, which also will be performed according to his/her will. Furthermore, although approximately one in three deceased are buried without a funeral within the Church of Sweden (Church of Sweden, 2023), Swedish newspapers still seem to have these funerals as the norm.

A comparison of the articulations of ‘funeral’ in different news media shows that the narratives of ‘funeral’ as a risk and as an essential ritual are primarily present in the tabloids Expressen and Aftonbladet. In contrast, ‘funeral as a profession’ is nearly exclusively present in the national newspapers SvD and DN. The distribution of the articles is not surprising, as the first two discourses are framed by dramatic language built on intense emotions, such as fear and love, a common way of building a story in tabloids. The professional discourse is less emotional and dramatic, thereby more in line with the national daily newspapers’ dramaturgy. However, ideology and power are present in all newspapers and shape readers' understanding of ‘funeral.’

## Conclusions

Analyzing articulations of a social phenomenon, such as ‘funeral,’ in crises, such as the Covid-19 pandemic, reveals norms that in other times might be hidden and, therefore, more challenging to grasp. The analysis outlines an image of the ‘responsible mourner.’ This ‘rational’ woman follows the funeral regulations and arranges a church funeral shortly after the death of a relative. The newspapers also present an image of the ‘good funeral’ in the physical presence of mourners, performed in a Lutheran church, according to the deceased’s will and in honor of the dead. The ‘bad funeral’ is the opposite of the ‘good funeral’ and is the dominating understanding of the pandemic funeral situation.

Increased use of intersectional perspectives is needed in future research to examine further how power structures intersect in media representation, often strengthening current power relations in society.

## Appendix 1

**Table:**

Titel	Newspaper	Date
Viruset tvingar fram nya rutiner för begravningar	Dagens Nyheter	2020-03-25
Solidaritet för alla	Expressen	2020-03-25
Flera personer smittade efter en begravning	Aftonbladet	2020-03-27
Ställer in av rädsla	Aftonbladet	2020-03-29
Familjen kan inte begrava pappan	Aftonbladet	2020-03-29
Åtgärder i förorten dröjde två veckor – trots dödslarm	Expressen	2020-03-31
Sörjande Malmöfamilj skickar hem kyrkogästerna	Sydsvenskan	2020-04-02
Marika sjöng för sin pappa när han somnade in	Expressen	2020-04-05
Nuri Kino: Ännu en kniv i våra hjärtan	Svenska Dagbladet	2020-04-10
Judiska äldreboendet har drabbats hårt av viruset	Svenska Dagbladet	2020-04-12
Birgit Falk dog på smittdrabbat äldreboende	Göteborgs-Posten	2020-04-17
Fler planerar för döden – nyregistreringarna har fördubblats	Dagens Nyheter	2020-04-19
Avlidna placeras i kylcontainrar	Svenska Dagbladet	2020-04-22
TV-profilen misstänkt sjuk i corona efter begravningen	Expressen	2020-04-22
Covid-19 har tagit sig in ipå tre av fyra äldreboenden	Dagens Nyheter	2020-04-24
Peter Wolodarski: Det handlar om människoliv, inte platåer	Dagens Nyheter	2020-04-26
Åke, 73, ville hjälpa till i busskaoset – smitades och dog	Aftonbladet	2020-04-26
Coronakrisen/Landet runt	Expressen	2020-04-27
En strålande vårdag – men något är fel, väldigt fel	Svenska Dagbladet	2020-04-28
“På begravningen nekar jag kramar – känner mig dum”	Svenska Dagbladet	2020-05-04
Viktigt att kunna ta avsked även när vi inte får samlas	Dagens Nyheter	2020-05-16
Claes Borgström död – var smittad av coronaviruset	Aftonbladet	2020-05-16
30 procent fler döda jämfört med förra året	Göteborgs-Posten	2020-05-22
Begravningarna ökade med 261 procent i april	Dagens Nyheter	2020-05-26
Det här är dödens nya logistik	Dagens Nyheter	2020-05-30
Coronaviruset har förändrat riksdagens vardag	Göteborgs-Posten	2020-05-31
Vanligt med livskris när en gammal förälder dör	Göteborgs-Posten	2020-06-07
“En begravning är nästan som direktsändning”	Dagens Nyheter	2020-06-17
“Hur nära dödshjälp har coronavården av äldre varit?”	Dagens Nyheter	2020-06-24
Kaoset på boendet – där nära hälften dog	Expressen	2020-07-02
“Vi har lovat att ha öppet varje dag oavsett vad”	Dagens Nyheter	2020-07-06
Att sända eller inte sända	Sydsvenskan	2020-07-12
Corona rev upp gruvsamhället	Expressen	2020-07-18
Stopp vid gränsen i 4 månader	Aftonbladet	2020-07-24
Björn Wiman: Så påverkar coronapandemin också livet efter döden	Dagens Nyheter	2020-07-19
“Hejdå, vi ses snart” – några timmar senare gravida Annas hjärta stannat	Expressen	2020-08-15
Skandal om vi inte får sörja våra döda	Aftonbladet	2020-11-20
Fler får närvara på begravningar	Svenska Dagbladet	2020-11-21
Klart: Max 8 får samlas	Sydsvenskan	2020-11-21
Corona kan drabba dig, tro inget annat	Aftonbladet	2020-11-27
Carl Philip och Sofia har corona	Aftonbladet	2020-11-27
Bekräftar: Sofia och Carl Philip coronasmittade	Expressen	2020-11-27
Svenska prinsparet har bekräftad covid-19	Göteborgs-Posten	2020-11-27
Kungen bröt mot sin egen coronaregel	Expressen	2020-11-29
Här samlas Börje Salming and 22 andra hjältar för att ta farväl	Expressen	2020-11-30
Sven Wolter 1934-2020	Expressen	2020-12-01
“Vi pratade begravning”	Aftonbladet	2020-12-03
Nyhetslistan	Aftonbladet	2020-12-13
Ett sista farväl – utan begravning	Aftonbladet	2020-12-14
Pandemin har inneburit att fler planerar för sin egen död	Sydsvenskan	2020-12-19
“Fler och fler dör och begravs ensamma”	Dagens Nyheter	2020-12-26
Polis ingrep mot trängsel vid en begravning: “Det blir dubbelmoral”	Expressen	2020-01-03
“Avstå från nyårsfirande för att skona sjukvården”	Dagens Nyheter	2020-12-31
Sjuksköterskan Thereses pappa dog av viruset	Expressen	2021-01-03
Shamsullah lurade döden – på väg tillbaka efter covid-19	Göteborgs-Posten	2021-02-01
Faran är absolut inte över, betonade sjukhuschef Harald Roos	Sydsvenskan	2021-02-28
Nu kan mor och dotter få träffas igen	Sydsvenskan	2021-03-04
Corona tog Allan – Nu lär sig Solvig att lyssna på tystnaden	Expreseen	2021-03-31
Alien: Den töntiga armbågshälsningen klarar jag mig utan	Dagens Nyheter	2021-05-03
Hoppet om normalitet – fem tecken i världen	Svenska Dagbladet	2021-05-04
“Joakim frågade mig via ögondatorn: ‘Var är alla?”	Svenska Dagbladet	2021-08-23
Hon har ständig beredskap för att hantera döden	Dagens Nyheter	2021-08-26
Caroline och Annike om sorgen efter pappa: “Fruktansvärd saknad”	Expressen	2021-09-27
Vaccinbevis på krogar och tåg får ris och ros	Sydsvenskan	2021-12-21
Läkaren: Inget är bra i det här läget	Svenska Dagbladet	2021-12-26
